# Preserved D-waves may not exclude distal motor deficits during cervicothoracic spinal cord tumor resection: A letter to the editor

**DOI:** 10.1016/j.cnp.2026.10.005

**Published:** 2026-10-14

**Authors:** Apratim Maity, Jacob Gluski, Ethan D.L. Brown, Aryaa Karkare, Aladine A. Elsamadicy, Randy S. D'Amico, Sheng-fu Larry Lo, Gabrielle Santangelo, Justin W. Silverstein DHSc, Daniel M. Sciubba

**Affiliations:** a Northwell, New Hyde Park, NY, United States of America; b Department of Neurosurgery, North Shore University Hospital, Manhasset, NY, United States of America; c Department of Neurosurgery, Lenox Hill Hospital, New York, NY, United States of America; d Department of Neurology, North Shore University, Manhasset, NY, United States of America; e Department of Neurology, Lenox Hill Hospital, New York, NY, United States of America

Dear Editor:

Intraoperative neurophysiological monitoring (IONM) is essential during cervicothoracic spinal cord tumor resection, where the proximity of critical motor pathways carries substantial risk of postoperative neurologic deficit (Kothbauer, 2007). Motor-evoked potentials (MEPs) provide real-time assessment of the functional integrity of the motor pathway from cortex to muscle, while direct waves (D-waves) reflect corticospinal tract (CST) conduction more specifically. D-waves are thus considered a more stable indicator of structural CST integrity (Olmsted et al., 2023). Prior studies have established that D-wave preservation predicts long-term motor recovery, even if MEPs attenuate intraoperatively. As a result, preserved D-wave signals have been widely interpreted as a reassuring sign that supports continued tumor resection (Park et al., 2025; Thomas et al., 2026).

However, this framework rests on the assumption that corticospinal tract integrity is sufficient to ensure functional motor output. In practice, the motor system is hierarchical, and MEPs reflect not only CST integrity, but also synaptic transmission within the spinal cord, anterior horn cell function, and distal motor unit integrity. Injury affecting these distal components may result in sustained postoperative weakness despite preserved D-wave signals. Although this physiological distinction between the two modalities is well established, it is infrequently illustrated in the clinical setting of cervicothoracic tumor surgery. This is particularly relevant at the cervicothoracic junction, as the C8-T1 roots contribute to intrinsic hand function and fine motor control. Injury at these levels can result in disproportionate functional disability (Pollock et al., 2014). In this letter, we report a retrospective series and a representative case that illustrate this limitation in clinical practice.

We retrospectively reviewed patients who underwent resection of intradural spinal cord tumors involving the cervicothoracic junction (including the T1 level) at two academic centers between 2022 and 2026. All of these cases included multimodal IONM with both transcranial MEPs and D-wave monitoring **(Supplementary Fig. 1)**. Seven patients met inclusion criteria. MEP attenuation was defined as a ≥ 50% reduction in amplitude from baseline, and D-wave preservation was defined as maintenance of ≥50% baseline amplitude without sustained decline. Postoperative motor function was assessed using Medical Research Council finger abduction strength, with immediate postoperative weakness defined as any decrease from preoperative baseline.

The median patient age was 47.1 years (IQR 32.4–59.3); four out of the seven patients were female. Tumor histopathologies included ependymoma (*n* = 2), high-grade glioma (*n* = 1, had dorsal rootlet sacrifice), hemangioblastoma (*n* = 1), meningioma (*n* = 1), primary melanoma (*n* = 1), and one of unknown pathology (Supplementary Table 1). D-waves were preserved throughout surgery in six of seven patients. Among these six, intraoperative MEP attenuation occurred in four, all of whom developed immediate postoperative hand weakness. In contrast, neither of the two patients with preserved MEPs and preserved D-waves developed postoperative weakness. Of the four patients with immediate weakness, two recovered to baseline strength at follow-up and two had persistent motor deficits **(Supplementary Fig. 2).** To further illustrate this discordant neuromonitoring pattern, we highlight one representative case below.

A female in her 20s initially underwent resection of an intramedullary cervical tumor spanning C5-C7. MEPs transiently declined during tumor manipulation but recovered to baseline; D-waves remained stable throughout, and the patient was neurologically intact postoperatively aside from bilateral 4th- and 5th-digit numbness attributable to dorsal column involvement during myelotomy. At one-year follow-up, tumor progression extending to the T1 level prompted re-operation. During this resection, MEPs declined again during tumor manipulation, but this time, failed to recover by the procedure's end; D-wave signals remained fully preserved **(**Fig. 1**)**. The tumor was of relatively soft consistency but had poor planes given the history of radiation and recurrence. Postoperatively, the patient developed new bilateral intrinsic hand weakness and right lower extremity weakness. At follow-up, lower extremity strength improved, but bilateral 4th- and 5th- digit weakness persisted. A postoperative MRI obtained at 2-years follow up from re-resection is shown in **Supplementary Fig. 3**. The temporal association between MEP attenuation during tumor manipulation and the subsequent distal hand deficit, despite stable D-wave recordings, suggested injury involving the segmental motor pathways distal to the corticospinal tract component assessed by the D-wave. However, because postoperative imaging and electrodiagnostic testing did not definitively localize the injury, involvement of the C8–T1 motor rootlets or anterior horn cells remains a proposed mechanism rather than a confirmed diagnosis.

**Fig. 1:**
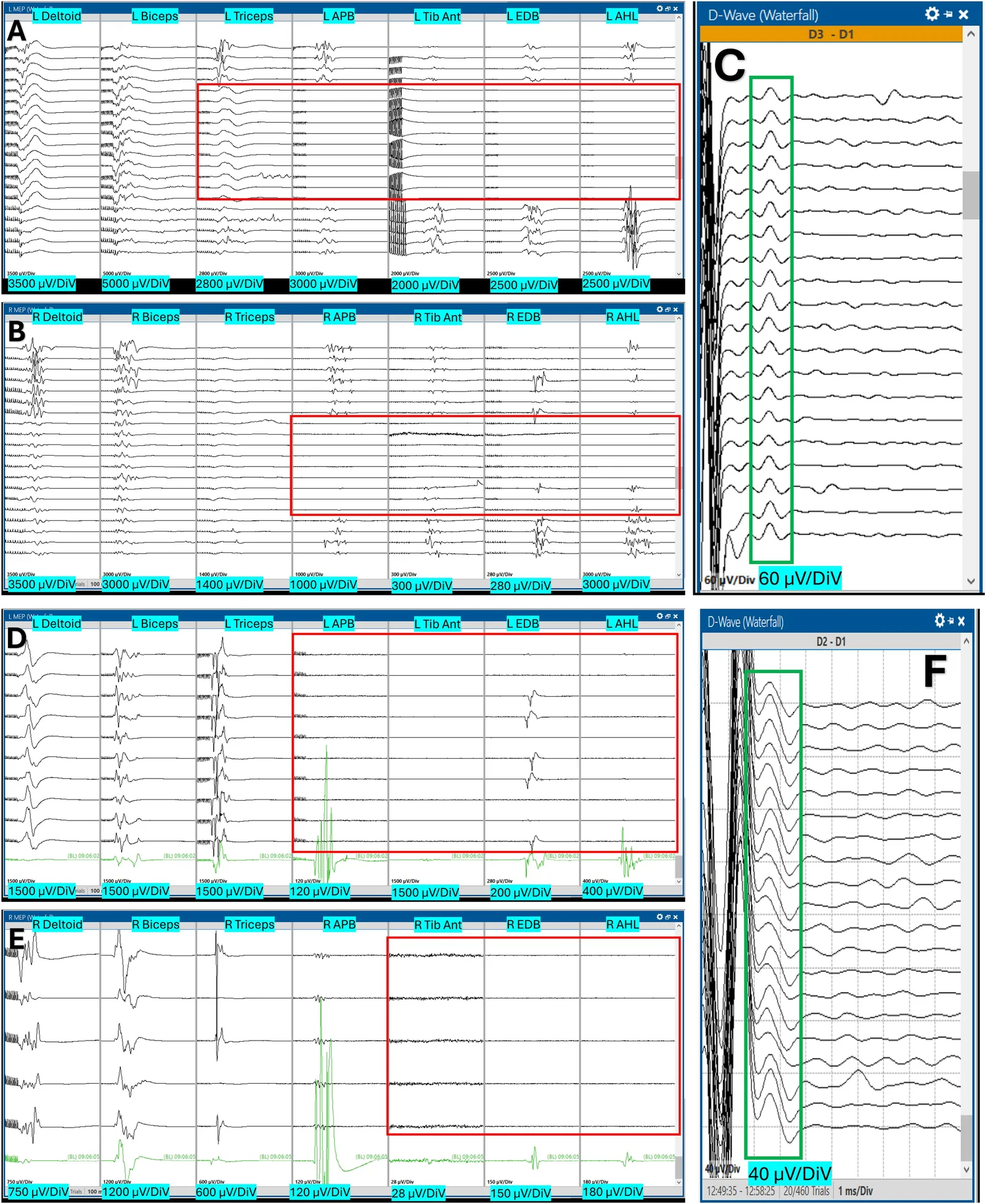
Intraoperative neuromonitoring recordings from the illustrative case demonstrating differing motor outcomes despite preserved D-wave recordings during two resections of the same intramedullary spinal cord tumor. (A–C) Recordings obtained during the initial resection of a C5–C7 intramedullary tumor. Muscle MEPs transiently attenuated during tumor manipulation but recovered to baseline by the conclusion of the procedure. Throughout the operation, subdural D-wave recordings remained stable with preserved latency, morphology, and amplitude. The patient awoke without new motor deficits. (D—F) Recordings obtained during repeat resection one year later for tumor progression extending to the T1 level. (D) Left-sided muscle MEPs demonstrated progressive attenuation with complete loss of distal responses, including the abductor pollicis brevis (APB), tibialis anterior (Tib Ant), extensor digitorum brevis (EDB), and abductor hallucis (AHL), while proximal upper extremity responses remained preserved. (E) Right-sided muscle MEPs demonstrated a similar pattern; the APB response underwent an approximately 99% reduction in amplitude but remained reproducible, whereas lower extremity responses were absent. (F) Subdural D-wave recordings obtained caudal to the lesion remained stable throughout the procedure, with preserved latency, morphology, and amplitude despite profound attenuation or loss of distal muscle MEPs. Postoperatively, the patient developed persistent bilateral intrinsic hand weakness with subsequent improvement in lower extremity strength. This serial comparison demonstrates preserved corticospinal tract conduction during both operations but markedly different motor outcomes, illustrating that stable D-wave recordings may coexist with clinically significant distal motor pathway dysfunction. Red boxes highlight the interval during which distal muscle MEPs became profoundly attenuated or absent while proximal upper extremity responses remained preserved. Green box highlights the corresponding D-wave recordings, which remained stable in latency, morphology, and amplitude throughout the same period of tumor manipulation. (For interpretation of the references to colour in this figure legend, the reader is referred to the web version of this article.)

Prior meta-analyses have demonstrated that irreversible MEP attenuation and D-wave attenuation (whether reversible or irreversible) are associated with postoperative motor deficits, whereas reversible MEP changes alone carry less predictive weight (Addanki et al., 2025). Furthermore, D-wave preservation has been shown to correlate with long-term motor recovery even in cases of intraoperative MEP loss (Park et al., 2025; Sala et al., 2006). In our series, however, preserved D-waves did not prevent lasting intrinsic hand weakness, a pattern compatible with dysfunction occurring beyond the territory sampled by the D-wave recording. This dissociation is not novel in principle: muscle MEPs and D-waves interrogate different components of the motor system, and their divergence is expected whenever injury occurs distal to the corticospinal tract. Because postoperative investigations were not standardized, the precise anatomical site and mechanism of injury could not be confirmed in each individual patient.

These two modalities assess fundamentally different aspects of the motor pathway. Muscle MEPs reflect activation of the motor cortex and synaptic transmission along the corticospinal tract to anterior horn cells, which in turn recruit multiple motor units to generate the compound muscle action potential recorded from the target muscles. D-waves, in contrast, arise from direct activation of the fast-conducting fibers of the corticospinal tract (Di Lazzaro and Ziemann, 2013; Patton and Amassian, 1954). Because the epidural D-wave records both corticospinal tracts together as a single combined signal, it can miss injury to one side, whereas muscle MEPs are recorded from each limb separately and still show which side is affected (Deletis and Sala, 2008). The D-wave also depends specifically on corticospinal conduction and does not sample synaptic transmission at the anterior horn cell or the distal motor unit. For these reasons, the relative significance of MEP and D-wave changes varies with the type of surgery and the anatomical site of injury (MacDonald, 2017). When a lesion involves both the intramedullary parenchyma and the exiting radicular components, as in the present case, the two modalities should be weighted differently rather than interpreted interchangeably. Neurophysiological changes are most informative when they prompt an anatomically based diagnosis of the site of injury rather than a nonspecific alert, which underscores the value of integrating multiple modalities—D-waves, muscle MEPs, and electromyography, with spinal mapping where applicable—rather than relying on any single method.

These observations are consistent with prior reports. Multimodal monitoring combining D-waves, muscle MEPs, and electromyography has been found to predict complex distal upper-extremity function more reliably than D-waves alone in high-cervical intramedullary tumor surgery (Siller et al., 2024), and metameric hand weakness has been described despite preserved D-waves and distal MEPs during treatment of a cervical vascular malformation (Sánchez Roldán et al., 2021). Our series extends these observations to cervicothoracic tumor resection and provides a clinical illustration of a dissociation that, although long recognized on physiological grounds, is infrequently demonstrated in practice.

These observations carry implications for intraoperative decision-making at cervicothoracic levels. When MEP attenuation occurs in the setting of preserved D-waves, the pattern may not be uniformly benign. MEP decline during traction, rotation, or manipulation near the root entry zone may raise concern for segmental motor pathway injury, even when D-wave signals remain stable. In such scenarios, preserved D-waves confirm CST integrity but do not exclude the possibility of persistent functional motor deficit. Accordingly, MEP attenuation should be interpreted as a localizing signal and integrated with both surgical anatomy and the specific intraoperative maneuver performed. Electromyography may further provide complementary information in this setting by detecting irritation or injury to segmental motor pathways, but its interpretation is limited by variable sensitivity and anesthetic dependence. Recognition of this monitoring pattern may improve functional outcomes by prompting earlier modification of surgical strategy when distal motor pathway injury is suspected.

In conclusion, preserved D-wave signals during cervicothoracic spinal cord tumor resection should not be interpreted as a guarantee of functional motor preservation. In our series, MEP attenuation despite preserved D-waves was consistently associated with immediate postoperative hand weakness, and the illustrative case suggests that this pattern may result in persistent deficit when injury occurs distal to the corticospinal tract. These observations underscore the need to interpret IONM data in the context of surgical anatomy and operative maneuver, and to recognize MEP attenuation (even in isolation) as a clinically meaningful signal at the cervicothoracic junction.

The following are the supplementary data related to this article.

**Supplementary Fig. S1:**
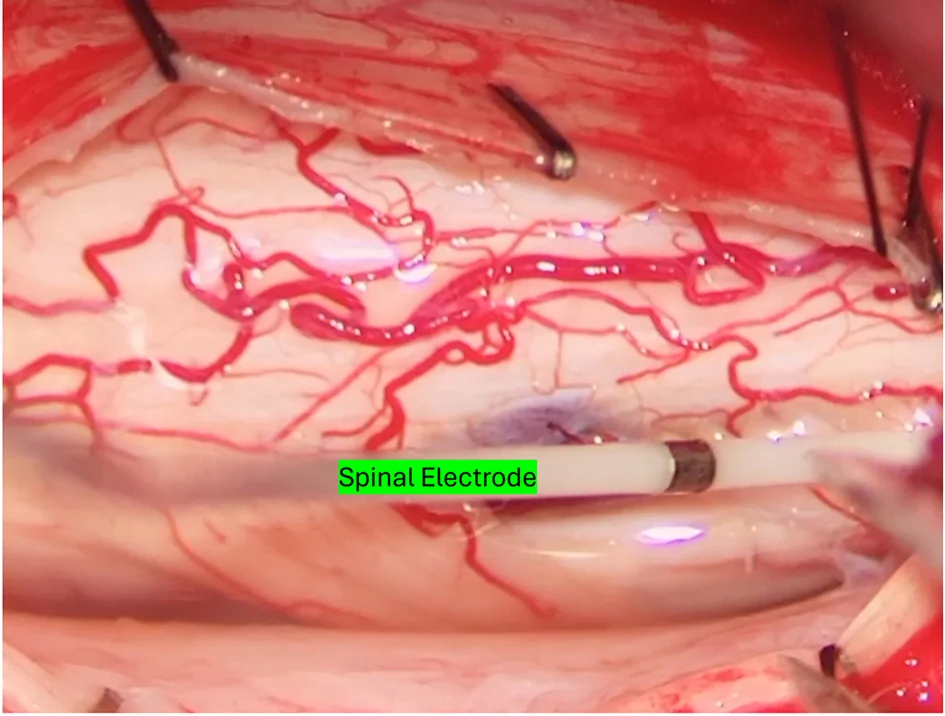
Epidural positioning of spinal electrode for recording of D-waves. Intraoperative view demonstrating epidural electrode positioning caudal to the tumor at the T1 level for recording corticospinal tract D-waves. Electrodes are typically placed both rostral and caudal to the lesion to monitor tract conduction across the operative field.

**Supplementary Fig. S3:**
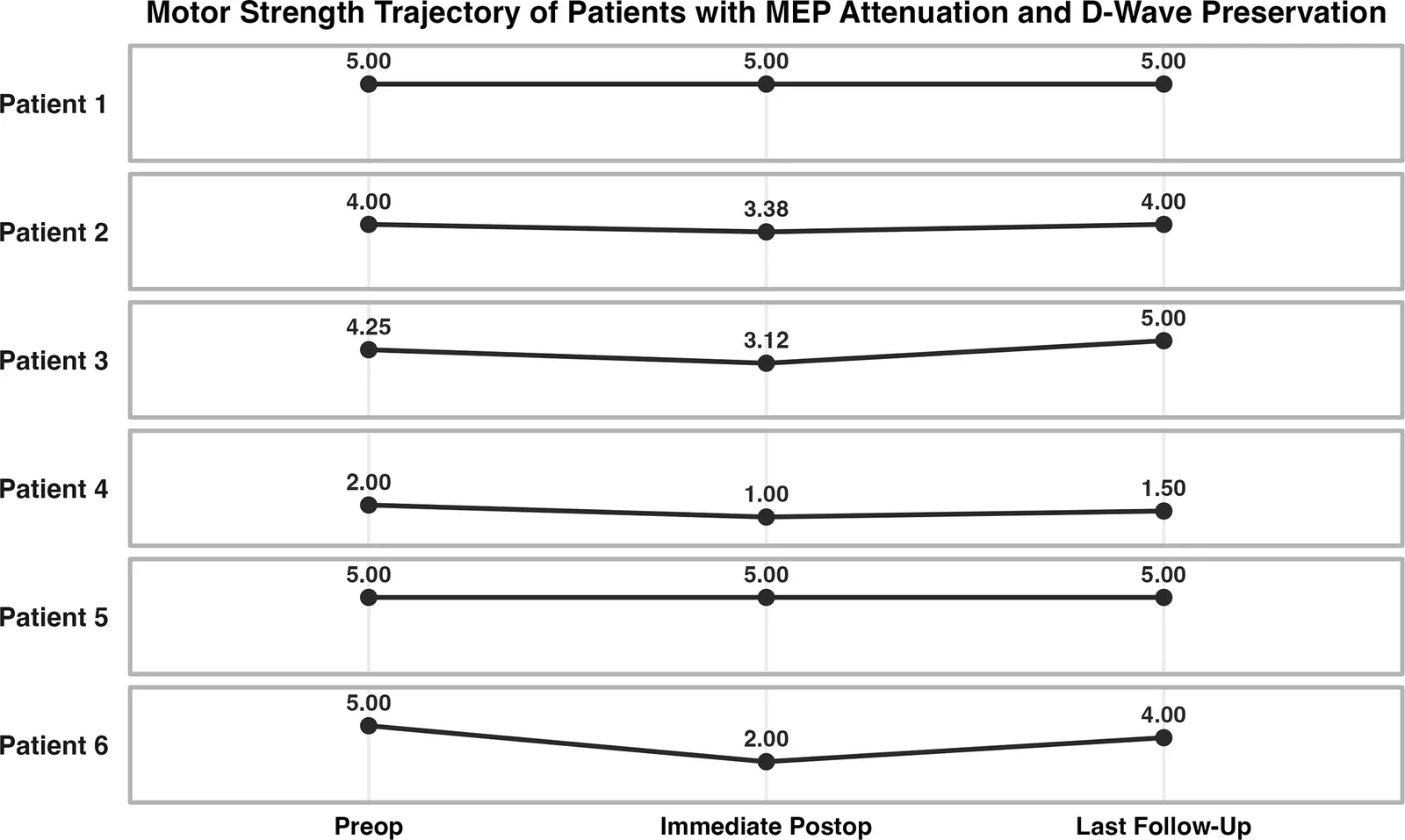
A line graph depicting individual patient trajectories of hand motor strength measured using the Medical Research Council (MRC) grading scale at preoperative baseline, immediate postoperative examination, and last clinical follow-up. For each time point, left and right finger abduction were averaged. The score “4-” was assigned a value of 4.0, “4” was assigned a value of 4.25 and “4+” was assigned a value of 4.75.

**Supplementary Fig. S3:**
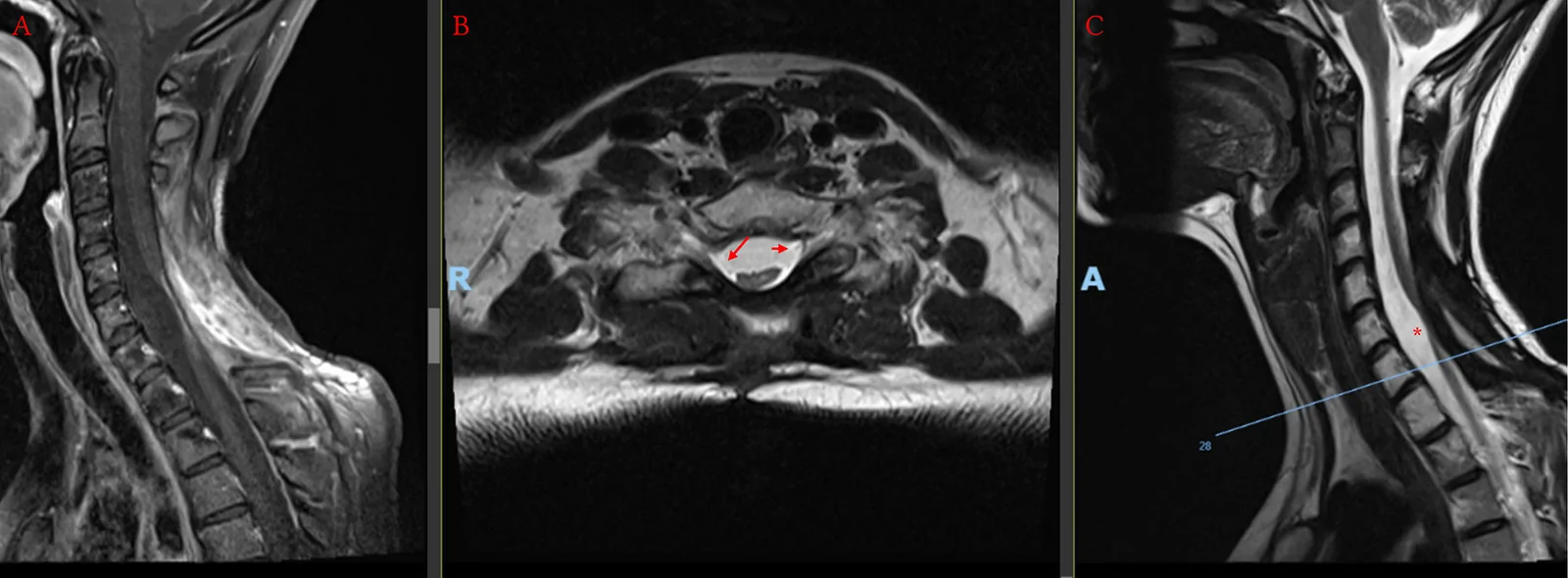
Cervical MRI obtained 2 years after the patient’s revision surgery. (A) Midsagittal postcontrast T1-weighted image showing fatty changes without tumor recurrence. (B) Axial T2-weighted image showing the spinal cord adherent to the posterior aspect of the thecal sac, with cord deformation related to the myelotomy. The arrows indicate the exiting T1 nerve roots, which appear grossly intact. (C) Midsagittal T2-weighted image. The blue line indicates the level of the axial section shown in panel B, and the asterisk indicates the site where the spinal cord is adherent to the dura.

Supplementary data to this article can be found online at https://doi.org/10.1016/j.cnp.2026.10.005.

## Declaration of competing interest

The authors declare the following financial interests/personal relationships which may be considered as potential competing interests: [The following disclosures do not represent conflicts of interest: Misters Maity, Brown, Ms. Karkare, Drs. Gluski, Santangelo, Elsamadicy and D'Amico have no disclosures. Dr. Lo discloses consulting for Depuy-Synthes, SpineAlign, Stryker, Bioventus, Moving Spine, Icotec, and Surgify Medical; Dr. Sciubba discloses consulting for J&J Medtech, Medtronic, Stryker, Baxter, Pacira, Icotec, SI-bone and royalties from J&J Medtech. Dr. Silverstein has an interest in Neuroprotective Solutions LLC and discloses consulting for IOTA Biosciences Inc].
